# Supplementary material for: New Prospects for Improving Microspore Embryogenesis Induction in Highly Recalcitrant Winter Wheat Lines
Source: Plants (Basel). 2024 Jan 25;13(3):363. doi: 10.3390/plants13030363 (PMC10857491; doi:10.3390/plants13030363)
Supplement: Supplementary file 1 [file plants-13-00363-s001.zip › plants-2768706-supplementary.pdf]

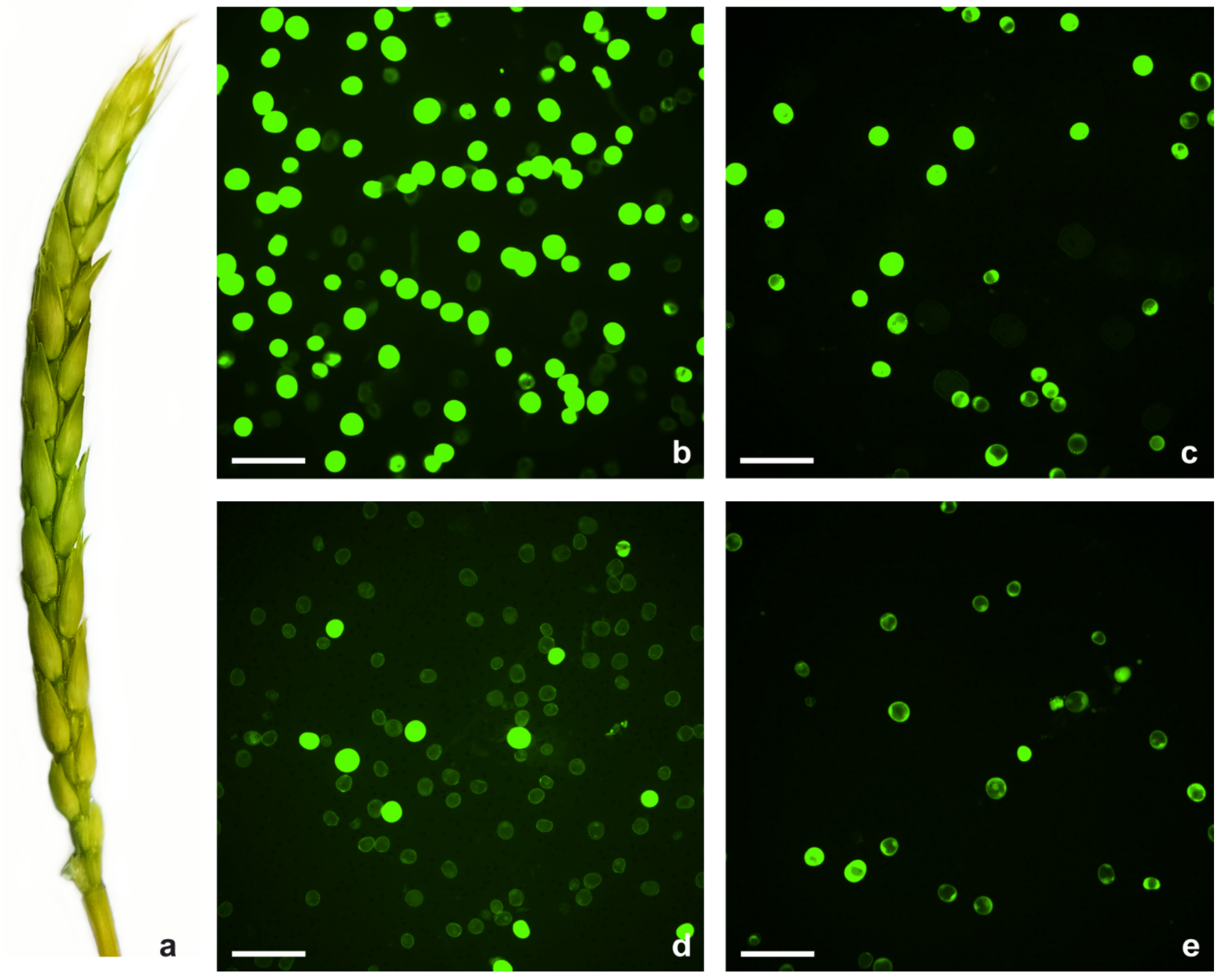

Figure S1. The effect of donor plant growth environment (controlled conditions compared to field-grown plants) on vitality of microspores isolated from spikes. Experiment 2020/2021. (**a**) Spike at the optimal phase for ME induction; (**b**, **c**) Microspores from plants grown under controlled conditions; (**d**, **e**) Microspores from field-grown plants. Microspores stained with FDA (fluorescence of vital cells intensely green). Bar = 200 µm.

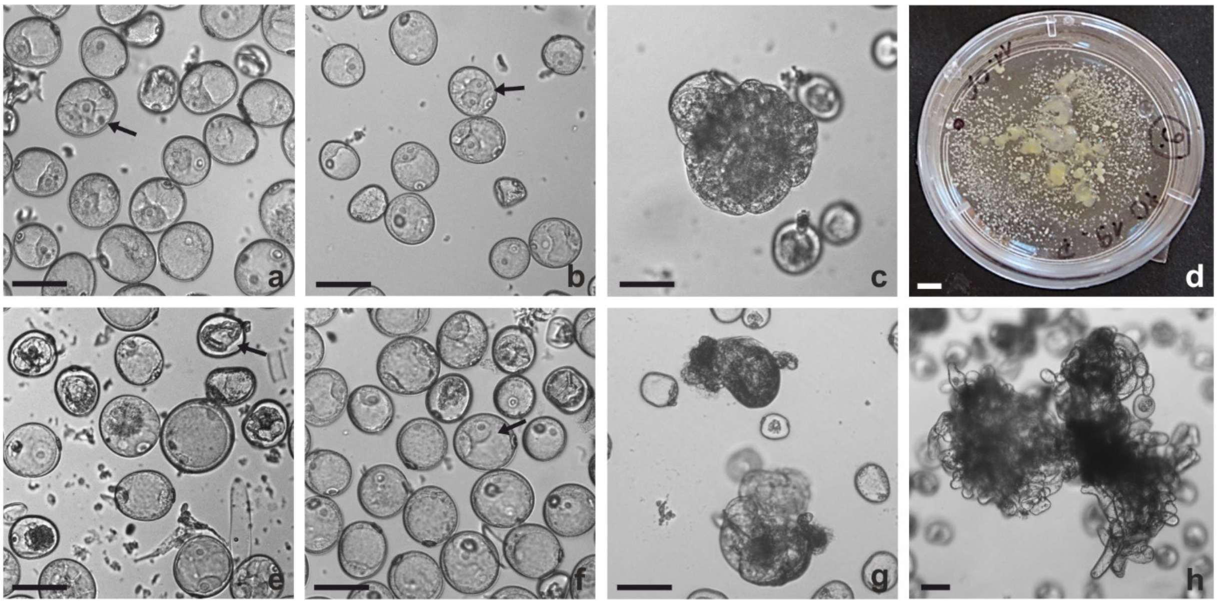

Figure S2. ME induction at 0, 14, 28 and 42d in suspension *in vitro* cultures of winter wheat (*Triticum aestivum* L.). (**a-d**) Properly developed embryo-like structures (ELS); (**e-h**) Suspension with low embryogenic potential to induce ELS with abnormalities.

(**a**) Microspores with star-like morphology (arrow points to SLS); (**b**) SLS after symmetric division (arrow); (**c**) Multicellular structure after 14d of culture; (**d**) Embryo-like structures (ELS) at the globular stage (0.5-1 mm in size) in co-culture with ovaries on Petri dish after 42 days of *in vitro* culture; (**e**) Microspore suspension after 14 days of culture (arrow points to dead microspore); (**f**) Microspores continuing gametophytic development in suspension; (**g**) Aborted multicellular structures; (**h**) Callus-like structure.

(**a**, **b**, **e**, **f**) Microspores suspensions on the day of isolation; (**c**, **g**) Microspore suspension after 14 days of culture; (**d**) Microspore suspension after 42 days of culture; (**h**) Microspore suspension after 28 days of culture; (**a**-**c**, **e**-**h**) Bar = 50 μm; (**d**) Bar = 2 mm.

Table S1. Frequency of ME induction [ELS/spike] and green plant regeneration [GR/spike] in microspore cultures of winter wheat breeding lines of F1 and F4 generations. Experiment 2020/2021.

(*) Data for field grown plants; ELS – embryo like structures; GR – green regenerants

Wheat line		Experiment 2020/2021	
		ELS/spike	GR/spike
CH1	F1	0	–
K20290	F1	0.9	0
K393	F1	0	–
SM IHAR	F1	0	–
MHR	F1	0	–
PO19	F4	16*	0
PO20	F4	2.0	0

(*) Data for field grown plants; ELS – embryo like structures; GR – green regenerants

Table S2. Frequency of ME induction [ELS/100 A] and green plant regeneration [GR/100 A] in anther cultures of winter wheat breeding lines of F1 and F4 generations. Experiment 202/2021.

Wheat line		Experiment 2020/2021			
		ELS/100A		GR/100A	
		$C17_{Fic}$	$C17_{Ph}$	$C17_{Fic}$	$C17_{Ph}$
CH1	F1	0.7*	0	0	–
K20290	F1	0.8*	0	0	–
K393	F1	2.0*	0	0	–
SM IHAR	F1	3.2*	0	0.8	–
MHR	F1	3.0*	0	2.0	–
PO19	F4	1.7*	0	0	–
PO20	F4	2.8*	0.2*	0.3	0

(*) Data for field grown plants; C17Fic - C17 induction medium supplemented with 50 g/dm3 Ficoll 400; C17Ph - C17 induction medium supplemented with 2.5 g/dm3 Phytagel

Table S3. Frequency of ME induction [ELS/spike] and green plant regeneration [GR/spike] in isolated microspore cultures of winter wheat breeding lines of F1 and F4 generations. Experiment 2021/2022.

Wheat line		Experiment 2021/2022	
		ELS/spike	GR/spike
CH1	F1	0	–
K20290	F1	0	–
K393	F1	0.7	0
SM IHAR	F1	0	–
MHR	F1	0	–
PO19	F4	0.1-0.7*	0.2
PO20	F4	0	–

(*) Data for field grown plants; ELS – embryo like structures; GR – green regenerants.

Table S4 Frequency of ME induction [ELS/100 A] and green plant regeneration [GR/100 A] in anther cultures of winter wheat breeding lines of F1 and F4 generations. Experiment 2021/2022.

Wheat line		Experiment 2021/2022											
		ELS/100A						GR/100A					
		$C17_{Fic}$			$C17_{Ph}$			$C17_{Fic}$			$C17_{Ph}$		
		10d	20d	30d	10d	20d	30d	10d	20d	30d	10d	20d	30d
CH1	F1	2.0	2.6	0.6	0.6	2.0	0	0	0	0	0	0	–
K20290	F1	4.0	2.6	0	0.6	2.0	0	2.0	0	–	0	0	–
K393	F1	0	0	0	0	0	0	–	–	–	–	–	–
SM IHAR	F1	0.6	0	0	0	0	0	0.6	–	–	–	–	–
MHR	F1	2.0	0	0	0.6	0	0	0	–	–	0	–	–
PO19	F4	3.4	0.6	0	0	0	0	0	0	–	–	–	–
PO20	F4	2.0	0.6	0	0	0	0	0	0	–	–	–	–

C17Fic- C17 induction medium supplemented with 50 g/dm3 Ficoll 400; C17Ph - C17 induction medium solidified with 2.5 g/dm3 Phytagel; 10d, 20d, 30d – Low temperature tillers pre-treatment for 10, 20 or 30 days at 4°C; A – anthers; ELS – embryo like structures; GR – green regenerants
